# Regulation of insect behavior via the insulin-signaling pathway

**DOI:** 10.3389/fphys.2013.00353

**Published:** 2013-12-03

**Authors:** Renske Erion, Amita Sehgal

**Affiliations:** Cell and Molecular Biology, University of PennsylvaniaPhiladelphia, PA, USA

**Keywords:** insulin signaling, *Drosophila melanogaster*, behavior, feeding, locomotion

## Abstract

The insulin/insulin-like growth factor signaling (IIS) pathway is well-established as a critical regulator of growth and metabolic homeostasis across the animal kingdom. Insulin-like peptides (ILPs), the functional analogs of mammalian insulin, were initially discovered in the silkmoth Bombyx mori and subsequently identified in many other insect species. Initial research focused on the role of insulin signaling in metabolism, cell proliferation, development, reproduction and aging. More recently however, increasing attention has been given to the role of insulin in the regulation of neuronal function and behavior. Here we review the role of insulin signaling in two specific insect behaviors: feeding and locomotion.

## Introduction

In both vertebrates and invertebrates, insulin is a key metabolic hormone that modulates carbohydrate and lipid metabolism in response to an organisms' nutritional state. Dysregulation of insulin production, release, or downstream signaling leads to metabolic disease, including diabetes and obesity (Baker and Thummel, 2007; Hoffmann et al., 2013). The role of insulin in metabolic homeostasis as well as development, fertility and lifespan is well-established, whereas the function of insulin in the brain and behavior is not as well-understood (Britton et al., 2002; Ikeya et al., 2002; Rulifson et al., 2002; Broughton and Partridge, 2009). In mammals, insulin is known to act on the brain to modulate behaviors relating to reproduction, feeding, and memory (Gerozissis and Kyriaki, 2003). However, given the complexities inherent to the study of mammals, the use of simpler organisms, such as insects, provides an excellent opportunity to elucidate the role of insulin signaling in neuronal function and behavior (Teleman, 2010).

Insulin-like peptides (ILPs) were originally discovered in the silkmoth *Bombyx mori* and subsequently identified in migratory locusts, mosquitos, and scarab beetles among other insects (Lagueux et al., 1990; Riehle et al., 2006; Lavine et al., 2013). The study of insulin signaling in insects was significantly advanced by the identification of eight insulin-like genes in the genome of the fruit fly, *Drosophila melanogaster* (Brogiolo et al., 2001; Grönke et al., 2010; Colombani et al., 2012). In mammals, insulin and insulin-like growth factors signal through several different receptors whereas in *Drosophila* all ILPs signal through a single insulin receptor (InR) (Fernandez et al., 1995; Yamaguchi et al., 1995; Brogiolo et al., 2001). In the adult fly, three of these ILPs (2, 3, and 5), are expressed in the dorso-medial protocerebrum by two clusters of neurosecretory cells known as the Insulin-Producing Cells (IPCs). The IPCs are located in the pars intercerabalis, a brain region analogous to the mammalian hypothalamus, and their axons project to other regions in the brain and to the fly heart (Rulifson et al., 2002). Secreted ILPs can activate insulin signaling in the brain or head or enter the fly circulatory system to activate systemic insulin signaling in peripheral tissues. The regulation of ILP production and release has recently been reviewed by Nässel et al. (2013).

In *Drosophila*, the field of behavioral genetics began with the identification of the *period* gene as a critical regulator of rest:activity rhythms in the fly (Konopka and Benzer, 1971). Since then the fruit fly has been used extensively to identify genes and pathways underlying many complex behaviors. This review will focus primarily on *Drosophila* largely because of its simple and well-studied neuroanatomy as well as its genetically tractable nature, both of which facilitate the study of insulin's role in the brain and complex behavior.

## The IPCs and insulin signaling pathway influence feeding behavior

Maintaining adequate energy stores is critical for animal survival and reproduction. As a result, neural and molecular mechanisms have evolved to modulate feeding behavior in response to an animal's internal physiological state as well as changes in food availability in the environment. Feeding begins with the motivation to eat, followed by a search for a suitable food source, which can be influenced by smell and taste among other factors. Feeding is terminated upon reaching a satiated state. In mammals it has long been known that insulin signaling suppresses food intake (Woods et al., 1979; Williams and Elmquist, 2012). Similarly, insulin has also been shown to play an important role at several stages of feeding behavior in insects, including *Drosophila melanogaster*. Studies in insects have also identified neuropeptides, many of which have mammalian orthologs, involved in the regulation of physiology and behavior, including feeding behavior (Nässel and Winther, 2010). Many of these other molecules and mechanisms regulating feeding behavior in fruit flies are reviewed by Itskov and Ribeiro (2013). Here we will focus solely only on instances in which insulin-signaling modulates *Drosophila* feeding behavior.

Hunger or deprivation due to limited food availability provokes animals to acquire food. Initiation of motivated foraging is crucial for survival so animals have developed mechanisms to ensure that this response occurs under appropriate conditions. The insulin signaling pathway links the fly's internal metabolic state with the initiation of feeding behavior. In larval IPCs, constitutive activation of a downstream effector of the InR, p70/S6 kinase (dS6K), reduces foraging motivation and food acquisition (Oldham and Hafen, 2003; Wu et al., 2005a). This attenuated feeding response may be mediated by increased ILP release since pan-neuronal overexpression of *ilp2* or *ilp4* also reduced motivated foraging (Wu et al., 2005a). Overall these data indicate that hunger normally downregulates S6K activity in the IPCs to reduce insulin release and drive deprived animals to search for and acquire food. Starvation-induced food acquisition is also modulated by insulin signaling in mushroom body neurons known as Kenyon cells (Zhao and Campos, 2012). Inhibition of insulin signaling in Kenyon cells throughout development reduces food intake following starvation. Interestingly, temporary suppression of synaptic transmission by these neurons also partially reduces food intake. Thus, insulin signaling in Kenyon cells during development may modulate the synaptic activity of these neurons to ultimately regulate food acquisition.

After assuming motivated foraging, animals must utilize their sense of smell in order to find an adequate food source. Starvation decreases the amount of time a fly takes to find food by heightening their sense of smell, or in more technical terms, by enhancing odor representation in neurons that process olfactory input, known as odorant receptor neurons (ORNs) (Root et al., 2011). Enhancement of odor representation is facilitated by the expression of short neuropeptide F (sNPF) and its receptor (sNPFR) in specific ORNs (Carlsson et al., 2010; Root et al., 2011). Both sNPF and its mammalian orthologue, Neuropeptide Y (NPY), are known to promote feeding behavior (Lee et al., 2004; Kageyama et al., 2012). Intriguingly, Root and colleagues found that insulin interacts with the sNPF pathway by acting as a satiety signal to decrease sNPFR expression in ORNs and in turn decrease motivated feeding (Root et al., 2011). Hence during starvation when insulin signaling is low, sNPFR levels are high to mediate increased ORN sensitivity to odors and to encourage food acquisition.

The gustatory system also plays a role in feeding behavior primarily by influencing food choice. When determining what to eat, animals must assess both the nutritional content and palatability of a food source. A hungry fly initially decides what to eat based on taste; choosing the sweeter option over the more calorie dense option. However, over time, this preference shifts toward the more calorie rich option (Stafford et al., 2012). This change in preference is at least partially mediated by insulin. *Ilp2* and *ilp3* mutants and decreased insulin signaling throughout the brain all show an increased initial preference for the more caloric food source instead of the sweeter option (Stafford et al., 2012). This suggests that insulin contributes to calorie sensing and food source preference by acting directly on neurons in feeding circuits.

In the absence of a preferred food source, animals will feed on less palatable food sources. Drosophila larvae prefer to feed on rich liquid food as opposed to solid food. This preference is partially mediated by insulin signaling in cells that promote food intake through signaling downstream of the receptor for NPF, a distinct peptide from sNPF but also a fly orthologue of NPY. Downregulating insulin signaling in NPFR neurons causes fed larvae to be hungry and also increases their consumption of the less preferred solid food (Wu et al., 2005b). Conversely, upregulation of the insulin pathway in NPFR neurons of deprived animals elicits attenuated feeding of solid food (Wu et al., 2005b). In states of starvation, animals adapt by undertaking risky behaviors including searching for food under less than optimal conditions and consuming normally aversive noxious food. Under unfavorable conditions like cold temperature, the NPF/IIS signaling cascade enables starving *Drosophila* larvae to adapt and continue searching for food in order to survive (Lingo et al., 2007). This pathway also regulates risk-sensitive food acquisition with respect to noxious or bitter compounds. Overexpression of NPFR as well as down regulation of insulin signaling in NPFR neurons both increase consumption of noxious or bitter compounds in non-deprived larvae (Wu et al., 2005b). These results indicate that the NPF and ILP signaling mediate foraging responses under adverse conditions presumably to promote survival.

After determining what to eat, animals must also determine how much they will eat. Under very poor nutrient conditions flies will compensate by eating more. Interestingly this change in feeding behavior does not occur in IPC ablated flies (Broughton et al., 2010). Similarly, inhibition of IPCs also results in attenuation of food intake under poor nutrient conditions (Cognigni et al., 2011). However, given that IPC ablation or inhibition presumably affects the expression levels of ILPs as well as other peptides expressed in these cells; alterations in feeding behavior may not simply be due to changes in insulin signaling. In addition to ILPs, the IPCs also express the cholecystokinin-like peptide, drosulfakinin (DSK) (Park et al., 2008; Söderberg et al., 2012). IPC specific reduction of *dsk* increases food consumption and alters the ability of the fly to discriminate between food choices. However, this effect is not independent of insulin because reduction of *dsk* also alters *ilp* transcript levels (Söderberg et al., 2012). Nonetheless, DSK likely modulates the amount of food a fly consumes by acting in conjunction with DILPs to convey fullness to the animal.

In addition to feeding behavior, *Drosophila* has also been used to investigate the mechanisms underlying physiological and behavioral responses to alcohol consumption (Devineni and Heberlein, 2013). The IPCs and insulin signaling in the brain are implicated in ethanol sensitivity and the behavioral response to this addictive drug. Inhibiting protein kinase A (PKA) activity in the IPCs increases ethanol sensitivity as does decreasing insulin receptor signaling suggesting that reduced PKA activity in IPCs results in decreased insulin production and/or release which ultimately causes increased ethanol sensitivity (Corl et al., 2005). On the other hand, exposure to ethanol can affect insulin signaling. Developmental ethanol exposure in the fly, as in many other organisms, has been shown to have detrimental effects. Flies that experienced developmental ethanol exposure are less viable, developmentally delayed, smaller in body size, and respond differently to alcohol as adults. These phenotypes are all due to reduced insulin signaling in exposed animals (McClure et al., 2011).

Some of the mechanisms involved in the regulation of feeding behavior discussed above have also been found to play a role in feeding behavior regulation in other insects not discussed here such as the African malaria mosquito *Anopheles gambia* and honey bees (Arsic and Guerin, 2008; Wang et al., 2010). Generally insulin conveys the organism's nutritional state, whether starved or fed, and accordingly drives feeding behavior in response to that state.

## Effects of insulin signaling on locomotor activity

Many insects, including *Drosophila*, are crepuscular; they are primarily active at twilight or dawn and dusk. An internal clock entrained to the external environment largely through light and temperature cues, tightly regulates the timing of locomotor activity [Reviewed in Allada and Chung (2010)]. This well-conserved molecular oscillator is comprised of interlocked transcriptional/translational feedback loops, in which clock proteins negatively regulate their own transcription to create an endogenous rhythm of roughly 24 h [Reviewed by Zheng and Sehgal (2012)]. Briefly, in *Drosophila*, the core molecular oscillator consists of two transcription factors, CLOCK (CLK) and CYCLE (CYC), which drive the expression of the clock genes *period* (*per*) and *timeless* (*tim*). Eventually PER and TIM proteins re-enter the nucleus and inhibit the activity of CLK/CYC, thus preventing their own transcription. Ultimately the inhibition of CLK/CYC is relieved following the degradation of PER and TIM, allowing the cycle to begin again.

The anatomical and functional organization of the roughly 150 clock neurons in each hemisphere of the adult fly brain is well-established (Allada and Chung, 2010). Clock neurons located within the ventral lateral regions of the brain, known as the small ventral lateral neurons (sLNvs), are sufficient for driving locomotor rhythms under conditions where no external cues are present (constant darkness and temperature). The sLNvs produce the neuropeptide pigment dispersing factor (PDF) which rhythmically accumulates in the dorsal projections of the sLNvs. These axonal projections terminate near the PI, the aforementioned neuroendocrine center that encompasses the IPCs among other neurosecretory cells. The PI is implicated in the control of circadian rest:activity rhythms; however, neither the IPCs or the ILPs produced by these cells have been reported to directly regulate rest:activity rhythms (Helfrich-Forster et al., 2000). Nevertheless, there is data implicating insulin signaling in modulating the sensitivity of the circadian clock to oxidative stress, as measured in response to the redox cycling agent paraquat (Zheng et al., 2007). Activation of the insulin receptor initiates a signaling cascade that results in the retention of the forkhead transcription factor FOXO in the cytoplasm (Puig et al., 2003). *Drosophila* FOXO mutants and, by extension, perhaps elevated insulin signaling (which leads to the deactivation of FOXO), display increased sensitivity to oxidative stress. Thus, the metabolic state of the fly can influence the clock to affect behavioral rhythms.

In contrast to mammals, circadian regulation of the endocrine system has not been as extensively investigated in insects (Bloch et al., 2013). Neurohormones are excellent candidates for conveying temporal information from neuronal pacemakers to peripheral tissues because they are released from the brain and circulate in the blood allowing them to reach distal tissues and cells. ILPs are one such potential time-conveying neurohormone, but whether they are indeed regulated by the circadian system has yet to be definitively established in insects. In the blood feeding bug, *Rhodnius prolixus*, brains have been found to release ilps with a 24 h rhythm *in vitro* (Vafopoulou and Steel, 2002). Additionally, IPC projections in *Rhodnius* are described as closely associating with the axonal projections of PDF positive neurons in the circadian system, but whether these associations are indicative of functional synapses has not yet been determined (Vafopoulou and Steel, 2012). In *Drosophila*, microarray studies suggest the insulin signaling pathway may be regulated by the circadian system through the circadian expression of *susi*, a negative regulator of Phosphatidylinositol-3 kinase (PI3K) activity (Claridge-Chang et al., 2001; McDonald and Rosbash, 2001; Ueda et al., 2002; Wittwer et al., 2005). Additionally, slowpoke binding protein (SLOB) is expressed in a circadian manner in the IPCs and *slob* mutants have altered insulin-signaling, however a function for SLOB in behavior is unclear (Jaramillo et al., 2004; Sheldon et al., 2011).

In addition to being under circadian control, rest:activity behavior is also under the control of the homeostatic system which regulates rest duration and quality. Short sleep duration and poor sleep quality are associated with metabolic dysfunction (Spiegel et al., 2009). In *Drosophila*, octopamine, the insect equivalent of norepinephrine, promotes wakefulness by binding to octopamine receptors on the cell membranes of IPCs (Crocker and Sehgal, 2008; Crocker et al., 2010). Given that the major output of the IPCs is ILPs, we asked whether octopamine signaling in the IPCs promotes wakefulness by modulating the insulin signaling pathway. We found that this is not the case as increasing octopaminergic signaling in a *ilp2-3* mutant background still results in decreased nighttime sleep similar to increasing octopaminergic signaling in a wildtype background (Erion et al., 2013). In addition, manipulations that either decreased or increased insulin signaling in the brain or fat body did not decrease sleep (Erion et al., 2013). These data do not rule out the possibility that sleep homeostasis is modulated by insulin signaling but does strongly suggest that it is not responsible for relaying the wake-promoting effects of octopamine.

Aspects of locomotor activity are sexually dimorphic in *Drosophila*. Males move more steadily compared to females as indicated by fewer activity/inactivity periods (or start/stop bouts), while the total distance traveled is similar for both sexes (Gatti et al., 2000; Belgacem and Martin, 2002). Restricted expression of the sex-determination transcription factor, *transformer* (tra), identified a subset of neurons in the PI, henceforth called feminizing cells (FCs), capable of feminizing the locomotor activity pattern of male flies (Gatti et al., 2000). The axonal projections of both FCs and IPCs terminate at the corpus cardiacum/corpus allatum (cc/ca) (Gatti et al., 2000; Ikeya et al., 2002). This gland synthesizes juvenile hormone (JH) which regulates many important processes in insects including metamorphosis, reproduction, and aging (Tu et al., 2005). JH and insulin have both been implicated in the sexual dimorphism of locomotor behavior. Feeding male flies an inhibitor of 3-Hydroxy-3-Methylgluaryl CoA Reductase (HMGCR), a key JH biosynthesis enzyme, feminizes their locomotor activity (Belgacem and Martin, 2002). HMGCR mutants and targeted reduction of HMGCR in the ca both abolish sexual dimorphism. Similarly, sexual dimorphism in locomotor activity is eliminated in insulin receptor mutants and flies with ablated IPCs. Interestingly, the insulin receptor is expressed in the ca and insulin receptor mutants have altered JH levels suggesting that there is a link between the insulin and JH pathways (Tatar et al., 2001; Belgacem and Martin, 2006). Furthermore, targeted reduction of the insulin receptor in the ca suppresses HMGCR expression in this tissue (Belgacem and Martin, 2007). Lastly, male mutants of *takeout*, a putative JH binding protein and known circadian output gene, also display feminization of their locomotor activity (Meunier et al., 2007). Thus, it appears that insulin, JH, and takeout may interact to regulate sexual dimorphism of locomotor behavior in flies; however their exact relationship remains unclear.

## Conclusion

**Figure 1:**
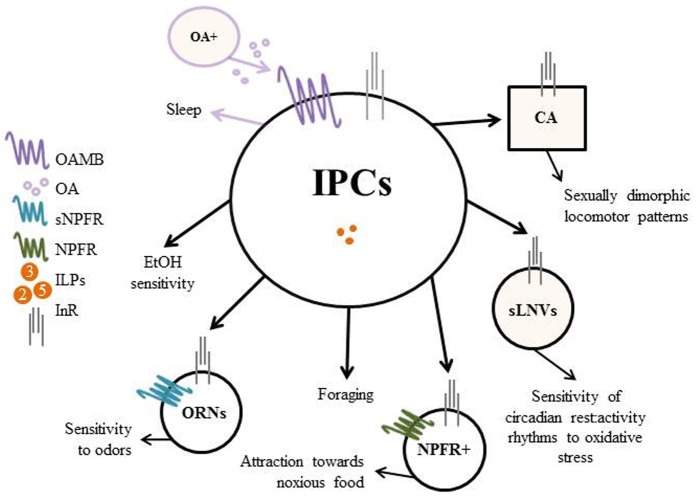
**Regulation of behavior by the Insulin-Producing Cells (IPCs) in Drosophila**. The IPCs modulate feeding and locomotor (shaded) behavior through the insulin pathway. Decreased insulin production/release from the brain IPCs results in decreased downstream insulin signaling and leads to increased ethanol (EtOH) sensitivity and motivated foraging. Decreased insulin signaling in sNPFR (short Neuropeptide F Receptor) expressing odor receptor neurons (ORNs) and in NPFR (Neuropeptide F Receptor) expressing neurons, increases the sensitivity of ORNs to odors and increases the attraction of flies towards normally aversive or noxious food sources, respectively. With respect to locomotion, octopaminergic neurons signal through the IPCs to promote wake; however, this effect is independent of insulin signaling. Insulin signaling in the corpus allatum (CA), a non-neuronal endocrine gland (indicated by square), drives sexual dimorphism of locomotor patterns. Lastly, insulin signaling in the circadian small ventrolateral neurons (sLNVs) modifies the sensitivity of rest: Lactivity rhythms to oxidative stress.

Given that behavior is so intricate, model organisms like *Drosophila melanogaster* as well as other insect species provide researchers with a framework to begin to teasing apart the many different pathways and molecules that contribute to behavior. In this review we have described the progress that has been made with respect to the regulation of feeding and locomotion by insulin, however many questions still remain (Figure 1). Future investigations will hopefully give us additional insight into the interactions between insulin and neural circuits responsible for regulating behavior.

### Conflict of interest statement

The authors declare that the research was conducted in the absence of any commercial or financial relationships that could be construed as a potential conflict of interest.
